# The Influence of Metabolic Syndrome in Predicting Mortality Risk Among US Adults: Importance of Metabolic Syndrome Even in Adults With Normal Weight

**DOI:** 10.5888/pcd17.200020

**Published:** 2020-05-21

**Authors:** Ting Huai Shi, Binhuan Wang, Sundar Natarajan

**Affiliations:** 1 New York University School of Medicine, New York, New York; 2 Veteran Affairs New York Harbor Healthcare System, New York, New York

## Abstract

**Introduction:**

Although metabolic syndrome (MetS) is less prevalent among normal-weight adults than among overweight and obese adults, it does occur. The objective of our study was to examine how mortality risks differed in weight categories stratified by presence/absence of MetS.

**Methods:**

We linked data for US adults responding to the National Health and Nutrition Examination Survey from 1999 through 2010 to data released from the National Death Index up to 2011. We grouped data according to categories of body mass index (normal [18.5 to <25.0 kg/m^2^], overweight [25.0 to <30.0 kg/m^2^], and obese [≥30.0 kg/m^2^]) and presence/absence of MetS. After conducting unadjusted analyses, we used Cox proportional hazards models to evaluate mortality risk as multivariable hazard ratios among obesity–MetS categories while controlling for selected covariates.

**Results:**

The analysis included 12,047 adults. The prevalence of MetS was 61.6% in the obese group, 33.2% in the overweight group, and 8.6% in the normal-weight group. The multivariate adjusted hazard ratio (95% confidence interval) for mortality among the obesity–MetS groups, compared with the normal-weight–no-MetS group, were as follows: normal-weight–MetS (1.70 [1.16–2.51]), overweight–no-MetS (0.99 [0.77–1.28]), overweight–MetS (1.10 [0.85–1.42]), obese–no-MetS (1.08 [0.76–1.54]), and obese–MetS (1.30 [1.07–1.60]); differences were significant only for the normal-weight–MetS group and obese–MetS group.

**Conclusion:**

MetS is a risk factor for mortality among normal-weight and obese adults. In our study, normal-weight adults with MetS had the highest mortality among the 6 groups studied, suggesting that interventions should also focus on MetS patients with normal weight.

**Summary**

**What is already known on this topic?**

Although studies show that metabolic diseases can affect normal-weight adults, studies on the risks of mortality have been equivocal and rarely focus on normal-weight adults.

**What is added by this report?**

We used national population-based survey data representative of the US population to compare groups of adults stratified by body mass index categories and presence/absence of metabolic syndrome. We found a higher risk of mortality among normal-weight adults with metabolic syndrome than among other groups without metabolic syndrome.

**What are the implications for public health practice?**

Although the prevalence of metabolic syndrome among normal-weight adults is low, it is associated with high risk of mortality. Because of the large number of normal-weight adults with metabolic syndrome at the population level, to prevent premature mortality, greater attention must be given to diagnosing and proactively treating metabolic syndrome in these normal-weight adults.

## Introduction

Although obesity is a well-known risk factor for poor metabolic health (1,2), metabolic health issues such as insulin resistance and diabetes risk also affect normal-weight people (3). A useful method for assessing metabolic health is to determine the presence of metabolic syndrome (MetS), which is defined as having 3 of the following 5 criteria: central obesity, elevated blood glucose, elevated triglycerides, low levels of high-density lipoprotein cholesterol, and elevated blood pressure (4).

Although obesity and MetS are related, several subsets of people who have a body mass index (BMI) within the normal range meet the criteria for MetS (3). An important area of study is the influence of MetS on clinical outcomes among people in various weight categories. Most studies of MetS have focused on obese people; little attention has been paid to normal-weight people, despite their risk of MetS and the complications it may portend. The risk of MetS among normal-weight people may be a more relevant public health problem now because of the increasing prevalence of MetS across all weight categories in recent years (5). Research that includes metabolically unhealthy normal-weight people shows equivocal results. Although several studies from around the world found an increased risk of diabetes or cardiovascular disease (6–11) among metabolically unhealthy normal-weight people, studies have not found an elevated risk of all-cause mortality in this group. Neither of 2 studies that used the US National Health and Nutrition Examination Survey (NHANES III) database stratified by MetS and BMI categories found significantly higher mortality in the group of adults with normal weight and MetS compared with a group of normal-weight adults without MetS (12,13).

To better understand the relationship between weight and MetS and how this relationship can be generalized to the US population, we used data from the 1999–2010 National Health and Nutrition Examination Survey (Continuous NHANES) to examine how mortality risks differ among groups of adults classified according to weight and presence/absence of MetS. We focused on people with normal weight and MetS. This information may help to refine prevention and treatment strategies among groups of people in various weight categories with and without MetS.

## Methods

### Study design and sample population

We used data from 1999–2010 NHANES. NHANES is a national publicly available database that has de-identified health and nutritional data on the US population. The data are compiled through surveys using interviews, physical examinations, and laboratory results. Participants are selected according to a complex sample design that clusters and stratifies the US population for the corresponding year. Some underrepresented groups are oversampled to provide more precise and reliable estimates. The sample was weighted to be representative of the US population for the given years using NHANES analytic guidelines for combining data across years (14). NHANES surveys are conducted continuously in 2-year intervals; from 1999 through 2010 (6 cycles), our study period, 62,160 people participated. Participants are interviewed about demographic, lifestyle, and health-related information. Medical and physiologic measurements are taken during a physical examination (15). We linked NHANES data with data from the National Death Index from 1999 to 2011; this database provides follow-up mortality data for up to 150 months for NHANES participants aged 18 or older (16). A minimum of 10 years is suggested for observing the effects of MetS on mortality (17).

We preliminarily excluded NHANES participants if they were younger than 20 years (n = 29,696). We then excluded participants if they had BMI less than 18.5 (n = 487); were missing data on education (n = 90), poverty-income ratio (n = 2,939), mortality follow-up (n = 49), MetS criteria (n = 18,183), or BMI (n = 2,472); or had a nonpositive survey weighting value (n = 1,934). In addition, we excluded participants with follow-up periods shorter than 12 months after the time of the survey to account for any frailty due to serious preexisting conditions (n = 648). The final analytic sample of 12,047 participants aged 20 to 85 had data for all variables examined in our study, eligible follow-up mortality data, and no preexisting frailty. NHANES collects data for people older than 85 but does not report these extreme values to protect privacy. 

Random subsampling accounted for most missing data points. Subsamples of participants were randomly selected to participate in various survey topics or laboratory testing. For example, less than one-third of all participants were tested for fasting glucose or triglycerides. Each subsample was further weighted so that each represents the US population for the given year. Less than 10% of missing data were due to nonresponse or refusals. Similarly, less than 10% of mortality data was lost at follow-up.

### Measurements

We categorized the study sample into 3 weight groups based on BMI according to standard definitions: normal weight (18.5 to <25.0 kg/m^2^), overweight (25.0 to <30.0 kg/m^2^), and obese (≥30.0 kg/m^2^). We further divided each weight group into 2 groups according to whether the participant met criteria for MetS. We defined MetS according to criteria provided by the American Diabetes Association, in which MetS is indicated by the presence of 3 or more of the following 5 criteria: central or abdominal obesity (men, >40 in waist circumference; women, >35 in waist circumference), triglycerides ≥150 mg/dL; high-density lipoprotein cholesterol (men <40 mg/dL; women <50 mg/dL), blood pressure ≥130/85 mm Hg, and fasting glucose ≥100 mg/dL (18).

We included the following covariates in the multivariate adjusted analyses: age (20–85), sex (male, female), race/ethnicity (non-Hispanic white, non-Hispanic black, Mexican American, other Hispanic, other/multiracial), education (<9th grade, 9th–11th grade, high school graduate, some college, college graduate), poverty-income ratio (0 to ≥5), smoking status (never smoked, former smoker, current smoker), and physical activity level (active, insufficiently active, and inactive). Physical activity level was defined by using categories proposed by Zhao et al (19), which were created according to physical activity guidelines published by the US Department of Health and Human Services (20) as follows: 1) physically active if they reported ≥150 minutes per week of moderate-intensity activity or ≥75 minutes per week of vigorous-intensity activity or ≥150 minutes per week of an equivalent combination (≥150 min/week); 2) insufficiently active if they reported some physical activity but not enough to meet the active definition (>0 to <150 min/week), or 3) inactive if they reported no (0 min/week) moderate-intensity or vigorous-intensity physical activity. Because of differences in questionnaires between NHANES cycles, we included only leisure-time physical activity in our analysis.

### Statistical analysis

Initial analyses using SAS complex survey frequency and means procedures that take into account weighting, stratification, and clustering of the data generated the descriptive statistics. We used the LIFETEST procedure to generate the unadjusted mortality data for each MetS–BMI category and the log rank test to determine significant differences between categories.

We evaluated the independent effect of obesity and MetS categories on mortality by using Cox proportional hazards models that accounted for the complex sampling design (weighting, stratification, and clustering), adjusted for age, sex, race/ethnicity, education, poverty-income ratio, smoking status, and physical activity to account for covariates commonly associated with mortality. Other important risk factors such as blood pressure, cholesterol, and blood glucose were already included in the definition of MetS. We excluded covariates, such as alcohol consumption, that did not significantly improve the statistical model. We used the 6-level BMI–MetS variable to find the hazard ratio of each group compared with the normal-weight–no-MetS group for all-cause mortality, cardiovascular mortality, and cancer mortality. The 6 groups were normal-weight–MetS, normal-weight–no-MetS, overweight–MetS, overweight–no-MetS, obese–MetS, and obese–no-MetS. We chose the normal-weight–no-MetS group as the referent because we hypothesized that it would be the healthiest. We then tested the moderating effect of BMI on MetS and mortality by testing the interaction between weight groups and MetS. To support the interaction analysis, we also tested the effect of MetS in each weight group, using the contrast statement to directly compare normal-weight–MetS participants and participants in other categories.

In a further analysis, while accounting for the complex sampling design and controlling for the same covariates, to determine the incremental influence of MetS on mortality, we compared each MetS group with their no-MetS counterparts in each BMI group. We performed all statistical analyses in 2017 using SAS version 9.4 (SAS Institute Inc), and a 2-sided *P* value <.05 was considered significant.

## Results

The prevalence of MetS was 61.6% in the obese group, 33.2% in the overweight group, and 8.6% in the normal-weight group. We found significant differences in the prevalence of MetS and weight groups for all demographic variables. Groups with MetS were generally older, less educated, and less physically active and had a lower income and a higher prevalence of smoking than their no-MetS counterparts (Table 1). Non-Hispanic white adults and other/multiracial adults had a lower prevalence of overweight and obesity but a higher prevalence of MetS compared with non-Hispanic black or Mexican American adults, whereas the inverse was true for Mexican American, other Hispanic, and non-Hispanic black adults.

**Table 1:** Baseline Characteristics of the Study Population (N = 12,047), by BMI and MetS Categories,^a^ Study of MetS and Mortality Risk, National Health and Nutrition Examination Survey, 1999–2010

Characteristic	Normal Weight (n = 3,223)		Overweight (n = 4,219)		Obese (n = 4,605)	
	No MetS	MetS	No MetS	MetS	No MetS	MetS
**No. (weighted %) of participants **	2,842 (28.4)	381 (2.7)	2,543 (22.8)	1,676 (11.3)	1,601 (13.4)	3,004 (21.5)
**Age, weighted mean (SE), y**	42.6 (0.5)	57.9 (1.1)	43.9 (0.4)	55.3 (0.5)	43.2 (0.6)	49.8 (0.4)
**Male sex, weighted %**	43.6	37.1	59.4	51.5	42.8	49.0
**Race/ethnicity, weighted %**						
Mexican American	5.7	5.4	9.2	8.5	8.9	7.9
Other Hispanic	3.2	6.4	5.5	5.4	5.9	3.3
Non-Hispanic white	75.0	75.1	70.9	76.3	63.5	73.6
Non-Hispanic black	8.5	4.8	9.8	5.6	18.1	11.1
Other or multiracial	7.6	8.2	4.6	4.2	3.7	4.1
**Education, weighted %**						
<9th Grade	4.7	9.4	5.4	11.1	6.5	7.0
9th–11th Grade	10.6	18.6	10.6	13.2	12.5	14.8
High school graduate	22.8	30.0	23.4	30.2	23.6	28.7
Some college	29.0	24.4	30.1	28.9	33.9	31.3
College graduate	32.9	17.7	30.6	16.6	23.5	18.2
**Poverty-income ratio,^b^ weighted mean (SE)**	3.2 (0.1)	2.6 (0.1)	3.2 (0.04)	3.0 (0.1)	3.0 (0.05)	2.9 (0.05)
**Smoking status, weighted %**						
Never	50.8	45.5	53.7	46.3	56.4	50.9
Former	20.6	27.3	25.4	32.3	26.1	28.8
Current	28.6	27.2	20.9	21.4	17.5	20.3
**Physical activity,^c^ weighted %**						
Inactive	32.2	48.2	32.2	47.5	40.8	47.9
Insufficiently active	22.2	19.8	22.8	22.0	22.1	24.4
Physically active	45.7	32.0	45.0	30.4	37.1	27.7

Abbreviations: BMI, body mass index; MetS, metabolic syndrome; SE, standard error.

a Based on standard definitions: normal weight (18.5 to <25.0 kg/m^2^), overweight (25.0 to <30.0 kg/m^2^), and obese (≥30.0 kg/m^2^). MetS defined as presence of ≥3 of the following 5 criteria: central or abdominal obesity (men, >40-in waist circumference; women, >35-in waist circumference), triglycerides ≥150 mg/dL; high-density lipoprotein cholesterol (men, <40 mg/dL; women, <50 mg/dL), blood pressure ≥130/85 mm Hg, and fasting glucose ≥100 mg/dL.

b Defined as the ratio of income to the federal poverty level.

c Physical activity level was defined by using categories proposed by Zhao et al (19), which were created according to physical activity guidelines published by the US Department of Health and Human Services (20).

**Figure 1:**
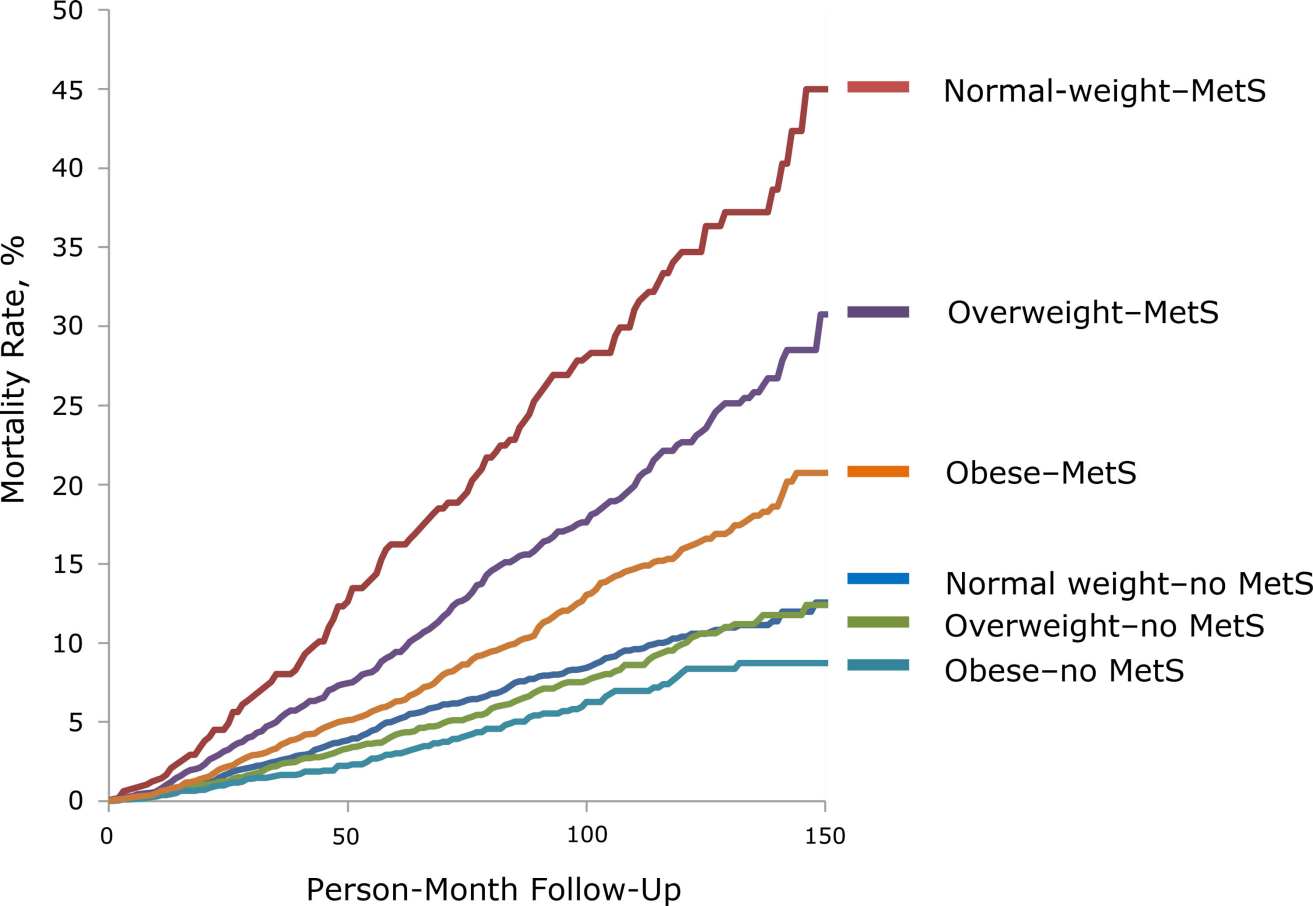
Unadjusted mortality curve during 150 person-month follow-up for each MetS–BMI category, National Health and Nutrition Examination Survey, 1999–2010, and National Death Index, 2011. Abbreviation: BMI, body mass index; MetS, metabolic syndrome.

According to the product-limit method from the LIFETEST procedure (Figure 1), the normal-weight–MetS group had the highest mortality rate. The log rank test showed significant differences between the normal-weight–MetS group and the overweight–MetS group (*P* <.001) and between the overweight–MetS group and the obese–MetS group (*P* <.001). Each no-MetS group had significantly lower mortality than their MetS counterparts, but we found no significant differences among no-MetS groups.

Follow-up by group ranged from 29,270 person-months (normal-weight–MetS group) to 221,490 person-months (normal-weight–no-MetS group) (Table 2). The number of deaths was lowest in the obese–no-MetS group (n = 61) and highest in the obese–MetS group (n = 255); the mortality rate per 1,000 person-month was lowest in the obese–no-MetS group (0.52) and highest in the normal-weight–MetS group (2.94). Unadjusted mortality rates showed that the normal-weight–MetS group had the highest mortality per person-month, followed by the overweight–MetS group and the obese–MetS group. Cox regression models that adjusted for age, sex, race/ethnicity, education, poverty-income ratio, smoking status, and physical activity showed significantly higher hazard ratios (HRs) for only the normal-weight–MetS group (HR, 1.70, 95% confidence interval [CI], 1.16–2.51) and obese–MetS group (HR, 1.30; 95% CI, 1.07–1.60) compared with the normal-weight–no-MetS group. The HR was higher in the normal-weight–MetS group than in the obese–MetS group, although the difference was not significant (HR, 1.31; 95% CI, 0.92–1.84). In the test of the interaction between weight groups and MetS, the *P* value for the interaction term in the full model was .16; however, tests for interactions typically have low power, and the recommended approach in causal inference is to base this on a priori hypotheses rather than the data. When we directly compared the normal-weight–MetS group with other groups, we found an HR of 0.64 (95% CI, 0.46–0.90; *P* = .01) in the overweight–MetS group and an HR of 0.76 (95% CI, 0.54–1.08; *P* = .13) in the obese–MetS group.

**Table 2:** Follow-Up, Mortality, and Association of MetS and BMI Status^a^ With Mortality (N = 12,047), National Health and Nutrition Examination Survey, 1999–2010, and National Death Index, 2011

Variable	Normal		Overweight		Obese	
	No MetS	MetS	No MetS	MetS	No MetS	MetS
Person months	221,490	29,270	195,434	129,018	117,742	220,715
No. (weighted %) of deaths	198 (4.1)	86 (19.4)	153 (3.8)	232 (9.8)	61 (3.4)	255 (6.7)
Mortality per 1,000 person months	0.89	2.94	0.78	1.80	0.52	1.16
Unadjusted hazard ratio (95% CI)	1.00 [Reference]	4.61 (3.20–6.64)	1.00 (0.80–1.25)	2.50 (1.92–3.25)	0.94 (0.65–1.35)	1.73 (1.40–2.13)
Age- and sex-adjusted hazard ratio (95% CI)	1.00 [Reference]	1.71 (1.19–2.46)	0.93 (0.75–1.15)	1.13 (0.88–1.45)	1.06 (0.76–1.46)	1.24 (1.02–1.52)
Multivariate adjusted hazard ratio^b^ (95% CI)	1.00 [Reference]	1.70 (1.16–2.51)	0.99 (0.77–1.28)	1.10 (0.85–1.42	1.08 (0.76–1.54)	1.30 (1.07–1.60)

Abbreviations: BMI, body mass index; CI, confidence interval; MetS, metabolic syndrome.

a Based on standard definitions: normal weight (18.5 to <25.0 kg/m^2^), overweight (25.0 to <30.0 kg/m^2^), and obese (≥30.0 kg/m^2^).

b Adjusted for age, sex, race/ethnicity, education, poverty-income ratio, smoking status, and physical activity.

**Figure 2:**
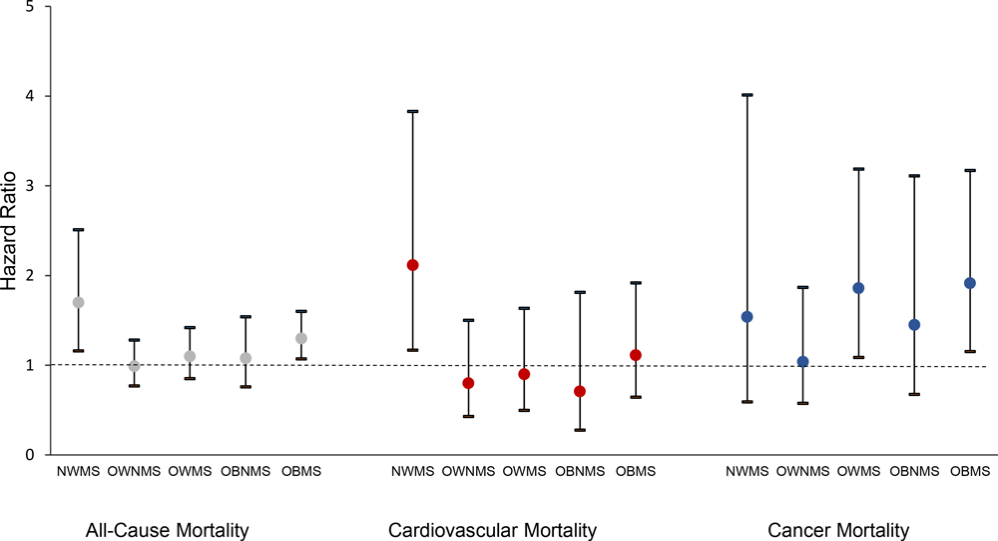
Weight–MetS categories and all-cause and selected cause-specific mortality, National Health and Nutrition Examination Survey, 1999–2010, and National Death Index, 2011. The normal-weight–no-MetS group was used as the reference group. Models were adjusted for age, sex, race/ethnicity, education, poverty-income ratio, smoking history, and physical activity. Error bars indicate 95% confidence intervals. Abbreviations: MetS, metabolic syndrome; NWMS; normal-weight–MetS; OWNMS, overweight–no MetS; OWMS, overweight–MetS; OBNMS, obese–no MetS; OBMS, obese–MetS.

In analyses of cause-specific mortality (Figure 2), we found that among 985 total deaths, 184 (weighted, 16.2%) were due to cardiovascular disease, 233 (weighted, 25.9%) were due to cancer, and the rest were due to causes that each accounted for less than 7% of total mortality. The adjusted Cox regression model for cardiovascular mortality showed a significant hazard ratio only for the normal-weight–MetS group (HR, 2.12; 95% CI, 1.16–2.51). The model for cancer mortality showed a significant hazard ratio for the overweight–MetS group (HR, 1.86; 95% CI, 1.09–3.19) and the obese–MetS group (HR, 1.91; 95% CI, 1.15–3.17).

**Table:** 

Group	All-Cause Mortality, Hazard Ratio (95% CI)	Cardiovascular Mortality, Hazard Ratio (95% CI)	Cancer Mortality, Hazard Ratio (95% CI)
Normal weight MetS	1.70 (1.16–2.51)	2.12 (1.17–3.83)	1.54 (0.59–4.01)
Overweight no Met S	0.99 (0.77–1.28)	0.80 (0.43–1.50)	1.04 (0.58–1.87)
Overweight Met S	1.10 (0.85–1.42)	0.90 (0.50–1.63)	1.86 (1.09–3.19)
Obese no MetS	1.08 (0.76–1.54)	0.71 (0.28–1.81)	1.45 (0.68–3.11)
Obese Met S	1.30 (1.07–1.60)	1.11 (0.64–1.92)	1.91 (1.15–3.17)
Normal weight no MetS	1 [Reference]	1 [Reference]	1 [Reference]

When we compared adults with MetS in each weight group with the no-MetS group (Table 3), we found that only the normal-weight–MetS group had a significant hazard ratio (HR, 1.70; 95% CI, 1.16–2.51). Although the obese–MetS group had a significantly higher HR when compared with the normal-weight–no-MetS group (HR, 1.30; 95% CI, 1.07–1.60), it did not have a significantly higher HR when compared with the obese–no-MetS group (HR, 1.20; 95% CI, 0.85–1.70).

**Table 3:** Evaluating the Incremental Effect of MetS on Mortality Risk by Comparing Adults With and Without MetS (N = 12,047), by Weight Status,^a^ National Health and Nutrition Examination Survey, 1999–2010, and National Death Index, 2011^b^

Model	Hazard Ratio (95% Confidence Interval) [*P* Value]		
	Normal Weight With MetS	Overweight With MetS	Obese With MetS
Unadjusted	4.61 (3.20−6.64) [<.001]	2.49 (1.94−3.20) [<.001]	1.84 (1.30–2.62) [<.001]
Age, sex adjusted	1.71 (1.19−2.46) [.004]	1.21 (0.94−1.56) [.13]	1.18 (0.85−1.63) [.32]
Multivariate adjusted^c^	1.70 (1.16–2.51) [.008]	1.11 (0.86–1.42) [.43]	1.20 (0.85–1.70) [.29]

Abbreviation: MetS, metabolic syndrome.

a Based on standard definitions: normal weight (18.5 to <25.0 kg/m^2^), overweight (25.0 to <30.0 kg/m^2^), and obese (≥30.0 kg/m^2^).

b The reference group for each hazard ratio is the counterpart without MetS in each weight category.

c Adjusted for age, sex, race/ethnicity, education, poverty-income ratio, smoking status, and physical activity.

## Discussion

When we evaluated mortality risk by obesity and MetS categories, only the normal-weight and obese groups with MetS had a significantly higher hazard ratio than the reference group, adults with normal-weight and no MetS. Although the normal-weight group had lowest prevalence of MetS, it also had the highest hazard ratio. Analysis of cardiovascular mortality showed a significantly higher hazard ratio only for the normal-weight–MetS group, which suggests a strong effect of MetS on cardiovascular death in normal-weight adults. Cancer mortality was significantly higher in the overweight–MetS and obese–MetS groups, compared with the normal-weight–no-MetS group, which is consistent with a previous study that found strong associations between adiposity and risk for many types of cancers (21).

The most likely explanation for the higher mortality in the normal-weight–MetS group is the influence of MetS through obesity-independent risk pathways. Although obesity is a known, common risk factor for MetS, MetS in the normal-weight population is likely due to factors independent of obesity. If these obesity-independent factors result in a more severe form of MetS, the normal-weight–MetS group would show a higher mortality rate than the obese–MetS group, whose mortality rate is attenuated by the less severe MetS caused by obesity. This phenomenon, sometimes known as a “collider stratification bias,” is cited as a possible cause of the obesity paradox, a phenomenon that similarly describes a lower mortality risk in obese patients with diabetes or cardiovascular disease (22).

Previous studies on normal-weight obesity attribute the cause of MetS in normal-weight adults to excess body fat percentage despite a normal overall weight. One study found that among participants with normal-weight BMI, those with a higher body fat percentage had a higher prevalence of abnormality in every MetS component (23). Normal-weight “obese” people have a BMI of less than 25 kg/m^2^, but they have symptoms of metabolic obesity, such as low insulin sensitivity, high hepatic fat, and high triglycerides. The location of adipose tissue can also affect metabolic health. Increased visceral adipose tissue accumulation is more strongly associated with risk of metabolic disorders than subcutaneous adipose tissue because of its location in the abdominal cavity and its large role in endocrine and inflammatory secretion (24). Therefore, the difference in adiposity of 2 people of similar weight can result in different susceptibility to insulin resistance and MetS, and this difference cannot be determined by BMI values.

Our findings do not mean that being obese with MetS is beneficial compared with being normal weight with MetS. Studies on the prognosis of overweight and obese patients show that risk of cardiovascular disease (25) inversely correlates with increasing weight (26). Other studies on the effect of weight loss show a significant improvement in all risk factors and symptoms of MetS after weight loss (27).

The results of 2 previous studies that examined mortality rates in MetS and BMI categories using NHANES III (1988–1994) data found results that are contradictory to the results of our study. Kuk and Ardern found higher HRs for the obese–MetS group, the obese–no-MS group, and the overweight–MetS group, but not for the normal-weight–MetS group when they used the normal-weight–no-MetS group as the reference (12). Durward et al found that only obese adults with MetS had a higher hazard ratio compared with the normal-weight–no-MetS group (13). However, although neither study found a significantly higher mortality risk in the normal-weight–MetS group compared with the normal-weight–no-MetS group, the HRs were 1.25 and 1.4, respectively. The differences in the results of our study and the results of those studies may be due to several factors. One, the NHANES III sample size was less than half of the Continuous NHANES sample size used in our study. Kuk and Ardern included 6,011 participants, and the Durward et al study included 4,373 participants, of whom only 77 were in the normal-weight–MetS group. The lack of significant differences in HRs between the normal-weight–MetS group and the normal-weight–no-MetS group in both studies was probably due to their small sample sizes. Two, the age-adjusted prevalence of MetS increased significantly from 29.2% in NHANES III (1988–1994) to 34.2% in Continuous NHANES (1999–2006) (5). Three, Kuk and Ardern included only 8 years of mortality follow-up data, resulting in a lower mortality rate than that observed in our 15 years of follow-up. Four, we used a more stringent definition of MetS than Kuk and Ardern used. Their definition excluded the criterion of central obesity and required only 2 conditions for diagnosis, which may have diluted the potent effect of MetS on all-cause mortality. Similar to our findings, Sahakyan et al found higher mortality in the normal-weight-with-central-obesity group than in their normal-weight-no-central-obesity group (11). Other studies that examined groups of people of primarily European descent stratified by BMI and metabolic health criteria — usually MetS or insulin resistance — found similarly higher risks for cardiovascular disease and diabetes in the normal-weight but metabolically unhealthy group (6–11), whereas 2 other studies found contradictory results (28,29).

The main strength of our study is the use of data from the Continuous NHANES, which rigorously measures all components of MetS, has data on a large number of people, and oversamples racial/ethnic minority groups, the elderly, and the poor. These weights, strata, and cluster variables were included in all analyses and allow our results to be generalizable to the US population. Therefore, in addition to usually studied population subgroups, our study included information on a large proportion of African Americans and Hispanic Americans. These populations are mostly absent in European studies, where normal-weight MetS was found to be a significant risk factor for cardiovascular disease and diabetes. NHANES also provides the statistical power necessary to answer the important question on the influence of MetS on normal-weight people. Furthermore, the Continuous NHANES is more recent and up to date than NHANES III, the database used in similar previous studies, so the data are more directly applicable to current clinical and public health practice.

Our study has several limitations. One limitation is the use of BMI to define weight groups. Many researchers argue that BMI is not a good indicator of weight status, because it does not differentiate among body compositions (26,30). Another limitation is that we lacked data from longitudinal time points. We have no information on how the characteristics of each participant, such as BMI, MetS, and other risk factors for mortality, changed from the time of the NHANES examination. The weight and metabolic condition of participants over multiple points as time-varying covariates would be needed to achieve a more comprehensive analysis than the one provided here.

Our study found that the normal-weight–MetS group had the highest risk of mortality among MetS and obesity categories — a risk that has not been previously identified in US adults. As such, greater attention must be given to normal-weight people who have MetS to provide early treatment and prevent future complications. In addition, the obese–MetS group had high mortality risk, particularly cancer mortality, when compared with the normal-weight–no-MetS group. Our study re-emphasizes that weight loss should continue to be encouraged among people who are obese.
